# Glasgow coma scale pupil score (GCS-P) and the hospital mortality in severe traumatic brain injury: analysis of 1,066 Brazilian patients

**DOI:** 10.1055/s-0043-1768671

**Published:** 2023-05-31

**Authors:** Melina Moré Bertotti, Evandro Tostes Martins, Fernando Zanela Areas, Helena Dresch Vascouto, Norma Beatriz Rangel, Hiago Murilo Melo, Katia Lin, Emil Kupek, Felipe Dal Pizzol, Alexandra J. Golby, Roger Walz

**Affiliations:** 1 Universidade Federal de Santa Catarina, Centro de Neurociências Aplicadas, Florianópolis SC, Brazil.; 2 Clínica Neuron, Florianópolis SC, Brazil.; 3 Hospital UNIMED, Departamento de Neurocirurgia, São José SC, Brazil.; 4 Hospital Governador Celso Ramos, Unidade de Terapia Intensiva, Florianópolis SC, Brazil.; 5 Hospital Universitário Polydoro Ernani de São Thiago, Departamento de Clínica Médica, Serviço de Neurologia, Florianópolis SC, Brazil.; 6 Universidade Federal de Santa Catarina, Departamento de Saúde Pública, Florianópolis SC, Brazil.; 7 Universidade do Sul de Santa Catarina, Laboratório Experimental de Patofisiologia, Programa de Pós-Graduação em Ciências da Saúde, Criciúma SC, Brazil.; 8 Hospital São José, Unidade de Terapia Intensiva, Criciúma SC, Brazil.; 9 Harvard Medical School, Brigham and Women's Hospital, Department of Neurosurgery, Boston MA, United States.

**Keywords:** Glasgow Coma Scale, Pupil, Brain Injuries, Traumatic, Prognosis, Mortality, Escala de Coma de Glasgow, Pupila, Lesões Encefálicas Traumáticas, Prognóstico, Mortalidade

## Abstract

**Background** Pupil reactivity and the Glasgow Coma Scale (GCS) score are the most clinically relevant information to predict the survival of traumatic brain injury (TBI) patients.

**Objective** We evaluated the accuracy of the GCS-Pupil score (GCS-P) as a prognostic index to predict hospital mortality in Brazilian patients with severe TBI and compare it with a model combining GCS and pupil response with additional clinical and radiological prognostic factors.

**Methods** Data from 1,066 patients with severe TBI from 5 prospective studies were analyzed. We determined the association between hospital mortality and the combination of GCS, pupil reactivity, age, glucose levels, cranial computed tomography (CT), or the GCS-P score by multivariate binary logistic regression.

**Results** Eighty-five percent ( *n* = 908) of patients were men. The mean age was 35 years old, and the overall hospital mortality was 32.8%. The area under the receiver operating characteristic curve (AUROC) was 0.73 (0.70–0.77) for the model using the GCS-P score and 0.80 (0.77–0.83) for the model including clinical and radiological variables. The GCS-P score showed similar accuracy in predicting the mortality reported for the patients with severe TBI derived from the International Mission for Prognosis and Clinical Trials in TBI (IMPACT) and the Corticosteroid Randomization After Significant Head Injury (CRASH) studies.

**Conclusion** Our results support the external validation of the GCS-P to predict hospital mortality following a severe TBI. The predictive value of the GCS-P for long-term mortality, functional, and neuropsychiatric outcomes in Brazilian patients with mild, moderate, and severe TBI deserves further investigation.

## Resumo

**Antecedentes** A reatividade pupilar e o escore da Escala de Coma de Glasgow (ECG) representam as informações clínicas mais relevantes para predizer a sobrevivência de pacientes com traumatismo cranioencefálico (TCE).

**Objetivo** Avaliar a acurácia da ECG com resposta pupilar (ECG-P) como índice prognóstico para predizer mortalidade hospitalar em pacientes brasileiros acometidos por TCE grave e compará-lo com um modelo combinando ECG e resposta pupilar com fatores prognósticos radiológicos.

**Métodos** Foram analisados dados de 1.066 pacientes com TCE grave de 5 estudos prospectivos. Foi determinada a associação entre mortalidade hospitalar e a combinação de ECG, reatividade pupilar, idade, níveis glicêmicos, tomografia computadorizada (TC) de crânio ou o escore ECG-P por regressão logística binária multivariada.

**Resultados** Oitenta e cinco por cento ( *n* = 908) dos pacientes eram homens. A média de idade foi de 35 anos e a mortalidade hospitalar geral foi de 32,8%. A AUROC (em português, Curva Característica de Operação do Receptor) foi de 0,73 (0,70–0,77) para o modelo utilizando o escore ECG-P e de 0,80 (0,77–0,83) para o modelo incluindo variáveis clínicas e radiológicas. O escore ECG-P mostrou acurácia semelhante na previsão da mortalidade relatada para pacientes com TCE grave derivados dos estudos *International Mission for Prognosis and Clinical Trials in TBI* (IMPACT, na sigla em inglês) e *Corticosteroid Randomization After Significant Head Injury* (CRASH, na sigla em inglês).

**Conclusão** Nossos resultados apoiam a validação externa da ECG-P para prever a mortalidade hospitalar após um TCE grave. O valor preditivo da ECG-P para mortalidade a longo prazo, resultados funcionais e neuropsiquiátricos em pacientes brasileiros com TCE leve, moderado e grave precisam ser investigados.

## INTRODUCTION

Traumatic brain injury (TBI) is a public health problem 1 that may result in death or permanent disability. 2 3 4 It represents a significant economic burden to society through high healthcare costs and lost productivity. 5 6 In 2016, there were 27 million new TBI cases worldwide, with > 60% of these in low- and middle-income countries. Recently estimated annual incidence per 100.000 inhabitants was 383 in Brazil, 333 in the USA, and 313 in China. 6 Santa Catarina, a southern Brazilian state with ∼ 7 million inhabitants in 2019, had 1,146 deaths related to TBI in the same year. Our recent prospective study in two metropolitan areas in the Santa Catarina state, with a combined population of 1,527,378, showed over 101.5 years of life lost per 100,000 inhabitants per year. 7 Unfortunately, the worldwide TBI incidence is rising, mainly due to the injuries associated with increased urban traffic and violence, leading to TBI being considered a “silent epidemic” 6 8 9 .

Many prognostic models have been developed to predict the outcome after TBI. 10 11 12 13 14 The corticosteroid randomization after a significant head injury (CRASH) trial (with 10,008 patients), which demonstrated that variables including Glasgow coma scale (GCS), pupil reactivity, the presence of significant extracranial injury, subarachnoid bleeding, and other abnormal results on computed tomography (CT), are all well-known prognostic factors. 14 15 Furthermore, the same research revealed that patients from low- and middle-income countries experienced higher mortality at 14 days than those from high-income countries, but a similar functional outcome at 6 months after trauma among the survivors. 14 Nevertheless, the use of prognostic scores that combine multiple risk factors has not found widespread acceptance in clinical practice because of a significant number of clinical measurements required. 15 16

In combination, the pupil reactivity and the GCS score are the most clinically relevant information to predict the survival of TBI patients. 11 12 15 17 18 To simplify the use of prognostic information in TBI, Brennan et al. proposed an arithmetic combination of the GCS score and pupillary response (GCS-P). 15 The GCS-P score was applied to the combined data from the CRASH study 19 and the International Mission for Prognosis and Clinical Trials in TBI (IMPACT) study with 11,989 patients, 18 and provided information about patient outcomes in comparison with more complex methods. 15 Although the CRASH study included a group of Brazilian patients ( *n* = 119), we aimed to assess the applicability of the GSC-P score in a large, prospective, and well-characterized sample of Brazilian patients.

The objective of the present work was to analyze the accuracy of the GCS-P score to predict the mortality during hospitalization in patients with severe TBI derived from five previous prospective studies carried out in the Santa Catarina state and compare the results with those from severe TBI patients in the combined CRASH 19 and IMPACT 18 data, using the same methodology as described by Brennan and colleagues. 15 Also, a comparison was made between the accuracy of the GCS-P model and the model with the GCS, pupil responsivity, and additional clinical and imagining data.

## METHODS

### Patients

The initial sample included 1,097 patients with severe traumatic brain injury from previous 5 prospective studies, 7 20 21 22 23 24 all of which were part of the Brain Trauma Database Project for the Santa Catarina state. The Ethics Committee for Research in Humans at the Federal University of Santa Catarina approved the project (Protocols 163/2005 of 2005 and 02832612.6.1001.0121 of 2013).

Patients were admitted to the hospital “Governador Celso Ramos” between January 1994 and December 2003 ( *n* = 748), and between April 2006 and September 2008 ( *n* = 83). The last 266 patients were admitted between April 2014 and January 2016 at the regional hospital of the city of Criciúma ( *n* = 61), the regional hospital of the city of São José ( *n* = 122), and the Hospital “Governador Celso Ramos” in the city of Florianópolis ( *n* = 83). These hospitals are the TBI reference centers that circumscribe the catchment area of over 1.5 million inhabitants in 2 metropolitan areas of the Santa Catarina state. Thirty-one patients (2.8%) were excluded because of the lack of pupil evaluation due to ocular trauma ( *n* = 12) or other missing variables ( *n* = 13), so that the final sample consisted of 1,066 patients. The inclusion criteria were a GCS score ≤ 8 or its deterioration within 48 hours of the TBI. The patients who evolved to brain death within 24 hours of admission were excluded from the present study. The primary endpoint was death during hospitalization so that the dependent variable was hospital mortality. The independent variables analyzed were age, sex, GCS score, cranial CT findings, glucose levels, and pupil reactivity at admission. Cranial CT findings were classified into six categories according to the Marshall classification. 25 26 The presence of traumatic subarachnoid hemorrhage was another independent variable. Computed tomography analysis was performed by one of the researchers and confirmed by the neurosurgeon when necessary, not blinded for the patient clinical status but always blinded for the patient outcome.

### Combining information about GCS score and pupil reactivity

We used the method reported by Brennan et al. 15 that combine a patient's GCS score and pupil findings into a single unidimensional index. First, we categorized pupils in the pupil reactivity score (PRS) according to the number of nonreactive pupils: if both pupils were unreactive to light, the score was 2, if only one pupil was unreactive to light, the score was 1, if both pupils were reactive to light, the score was 0. The GCS-pupil (GCS-P) score was obtained by subtracting the PRS from the GCS total score: GCS-P = GCS - PRS.

Another modification tested as a prognostic factor of hospital mortality among severe TBI patients was based on a previous study 24 that showed about a sixfold increase in mortality among the patients with bilateral mydriatic compared to anisocoric pupils at admission. A modified GCS-P proposed by the present study authors scored 3 instead of 2 in the Brennan et al. scheme. 15 If only one pupil was unreactive to light; the score was 1; if both pupils were reactive to light, the score was 0. The modified GCS-pupil score was obtained by subtracting the PRS from the GCS total score.

### Statistical analysis

Bivariate associations between the hospital mortality and the independent variables were analyzed by binary logistic regression, and the results were expressed as odds ratio (OR) with its 95% confidence interval (CI). The independent variables with significance level < 0.20 in the bivariate regression were included in a multivariate binary regression using the stepwise selection criterion. The Hosmer-Lemeshow test was used to evaluate the goodness of fit of the final model.

The area under the receiver operating characteristic (ROC) curve, abbreviated as AUROC, and its 95%CI, were used to assess the classification performance of the models under comparison. Split-half cross-validation was used to avoid fitting and testing classification performance on the same sample.

## RESULTS

Eighty-five percent ( *n* = 908) of the patients were men. The mean age was 35 years old, and the overall hospital mortality was 32.8%. The most frequent cause of TBI were road accidents (76.3%), followed by falls (15.1%), assaults (4.5%), firearm injuries (1.2%), and others (3%). The characteristics of survivors and nonsurvivors are shown in Table 1 . Mortality was associated with older age, higher glucose levels, Marshall CT classification injury type > II, traumatic subarachnoid hemorrhage on CT, lower GCS scores on hospital admission, and anisocoric or mydriatic pupils ( Table 2 ). The association between female sex and mortality shown in the univariate analysis ( Table 1 ) was not confirmed by the multivariate binary logistic regression ( *p* = 0.24) and this variable was not included in Table 2 .

**Table 1:** Bivariate logistic regression analysis for the association of mortality during hospitalization with demographic and clinical risk factors among the patients with severe traumatic brain injury

Predictive variables		All Patients N = 1,066 (%)	Outcome		Crude OR (CI 95%)	*P* -value
			Survivors n = 716 (%)	Non-survivors n = 350 (%)		
**Sex**	Male	908 (85)	624 (68.7)	284 (31.3)	1.0	
	Female	158 (15)	92 (58.2)	66 (41.8)	1.56 (1.11–2.207)	0.01
**Age (years old)**	Mean (±SD)	35.18 (16.52)	34.26 (15.92)	37 (17.56)	NA	
	12–30	530 (49.6)	368 (69)	164 (31)	1.0	
	31–45	264 (25.4)	181 (66.5)	91 (33.5)	1.12 (0.82–1.54)	0.44
	46–60	156 (14.8)	108 (68)	51 (32)	1.06 (0.72–1.55)	0.76
	> 60	109 (10.2)	62 (57)	47 (43)	1.70 (1.11–2.59)	0.01
**Glucose**	Mean (±SD)	160.4 (63.5)				< 0.0001
	≤ 110	148 (15.0)	110 (74.3)	38 (25.7)	1.0	
	111–220	721 (73.0)	495 (68.7)	226 (31.3)	1.32 (0.88–1.97)	0.17
	221–300	85 (8.6)	44 (51.8)	41 (48.2)	2.70 (1.54–4.74)	< 0.001
	> 300	33(3.3)	13(39.4)	20(60.6)	4.45 (2.02–9.81)	< 0.0001
**Marshall cranial CT classification**	Type I injury	93 (8.8)	79 (84.9)	14 (15.1)	1.0	
	Type II injury	239 (22.4)	200 (83.7)	39 (16.3)	1.1 (0.56–2.13)	0.77
	Type III injury	274 (25.7)	173 (63.0)	101 (37.0)	3.29 (1.77–6.11)	< 0.001
	Type IV injury	110 (10.3)	40 (36.4)	70 (63.6)	9.87 (4.96–19.65)	< 0.001
	Type V injury	300 (28.3)	197 (65.6)	103 (34.4)	2.95 (1.59–5.46)	0.001
	Type VI injury	44 (4.1)	22 (50)	22 (50)	5.64 (2.48–12.81)	< 0.001
**SAH**	No	641 (60.6)	464 (72.4)	177 (27.6)	1.0	
	Yes	417 (39.4)	248 (59.5)	169 (40.5)	1.78 (1.37–2.32)	< 0.001
** GCS ^a^**	8	211(19.9)	175(81.8)	39(18.2)	1.0	
	7	224(20.9)	187(83.1)	38 (16.9)	0.9(0.55–1.5)	0.71
	6	177(16.6)	135 (75.8)	43 (24.2)	1.43(0.87–2.32)	0.15
	5	60 (5.6)	33 (55.0)	27 (45.0)	3.67(1.98–6.8)	< 0.001
	4	151 (14)	70 (46.4)	81 (53.6)	5.2(3.24–8.3)	< 0.001
	3	243 (23)	122 (49.4)	125 (50.6)	4.6(2.99–7.05)	< 0.001
** Pupils ^b^**	Isochoric	535 (50.2)	433 (80.9)	102 (19.1)	1.0	
	Anisocorics	422(39.6)	259 (61.4)	163 (38.6)	2.68 (2.0–3.58)	< 0.00001
	Mydriatics	109 (10.2)	24 (22.0)	85 (78.0)	15.0 (9.10–24.8)	< 0.00001
** TBI Center ^c^**	Criciúma	61 (6.1)	39 (63.9)	22 (36.1)	1.0	
	São José	122 (12.1)	88 (72.1)	34 (27.9)	0.68 (0.36–1.32)	0.26
	Florianópolis	820 (81.8)	547 (66.5)	273 (33.5)	0.88 (0.51–1.52)	0.65

Abbreviations: CI, confidence interval; CT, computed tomography; GCS, Glasgow Coma Scale; OR, odds ratio; SAH, subarachnoid hemorrhage; TBI, traumatic brain injury.

Notes: ^a^ GCS at admission; ^b^ pupils reactivity at admission; ^c^ cities were the TBI reference centers are located.

**Table 2:** Multivariate binary logistic regression for the association of mortality during hospitalization with demographic and clinical risk factors among 1,066 patients with severe traumatic brain injury

Independent variables		Probability of death	95% CI bounds	
			Lower	Upper
**Age (years old)**	12–30	0.31	0.3	0.32
	31–45	0.32	0.30	0.32
	46–60	0.33	0.29	0.38
	> 60	0.42	0.38	0.46
**Glucose (mg/dl)**	≤ 110	0.31	0.23	0.39
	111–220	0.33	0.31	0.34
	221–300	0.39	0.36	0.42
	> 300	0.39	0.38	0.40
**Marshall cranial CT classification**	Type I injury	0.24	0.17	0.30
	Type II injury	0.23	0.17	0.29
	Type III injury	0.34	0.3	0.37
	Type IV injury	0.57	0.50	0.63
	Type V injury	0.30	0.28	0.31
	Type VI injury	0.56	0.55	0.56
**Glasgow Coma Scale**	3	0.43	0.38	0.49
	4	0.46	0.42	0.50
	5	0.45	0.39	0.50
	6	0.25	0.24	0.26
	7	0.20	0.17	0.24
	8	0.24	0.22	0.27
**Pupil reactivity**	Isochoric	0.22	0.19	0.26
	Anisocoric	0.37	0.34	0.40
	Mydriatic	0.63	0.50	0.75
**SAH**	No	0.28	0.27	0.29
	Yes	0.39	0.38	0.40

Abbreviations: CI, confidence interval; CT, computed tomography; SAH, subarachnoid hemorrhage.

Split-half cross-validation model showing an average sensibility of 76.9% (range 74.7–79.2%), a specificity of 63.1% (61.5–64.8%) for this model. The Hosmer & Lemeshow goodness-of-fit test produced the Pearson chi-square of 392 with 387 degrees of freedom and associated *p* -value of 0.418, thus confirming a good fit of the final model.

For comparison, the proportion of patients with severe TBI according to the GCS score in the IMPACT/CRASH combined data bank, relative to the present study sample, is shown in Table 3 . The proportion of deaths at 6 months after the hospitalization of patients from the IMPACT/CRASH data bank ( *n* = 9,057) was 33.9%, similar to the 32.8% observed in the present study. The GCS score decline was also associated with increased mortality in the studies under comparison ( Table 3 ).

**Table 3:** Mortality of patients from the CRASH/IMPACT sample at 6 months after traumatic brain injury, the mortality during hospitalization in the present study according to the GCS-P score and the association between the GCS-P score and the hospital mortality.

Combined CRASH/IMPACT data (n = 9,153)			Present study (n = 1,066)			Binary regression ^a^	
GCS-P score	*n* (%)	Mortality at 6 months after TBI (%)	GCS-P score	*n* (%)	Hospital mortality (%)	Crude OR (95%CI)	*p* -value
8	1073 (11.7)	20.0	8	143 (13.4)	16.0	1.0	
7	1930 (21.1)	19.2	7	188 (17.6)	12.2	0.72 (0.39–1.35)	0.32
6	1550 (16.9)	25.0	6	185 (17.3)	20.0	1.3 (0.73–2.31)	0.36
5	1136 (12.4)	32.6	5	111 (10.4)	34.2	2.71 (1.5–4.91)	< 0.001
4	1016 (11.1)	39.5	4	80 (7.5)	37.5	3.1 (1.65–5.9)	< 0.0001
3	1178 (12.9)	40.9	3	197 (18.5)	45.7	4.3 (2.59–7.43)	< 0.0001
2	636 (6.9)	64.6	2	112 (10.5)	58.9	7.48 (4.1–13.4)	< 0.0001
1	634 (6.9)	74.4	1	50 (4.7)	86.0	32 (12.83–80)	< 0.0001

Abbreviations: CI, confidence interval, GCS-P, Glasgow coma scale pupil score; OR, odds ratio; TBI, traumatic brain injury.

Note: ^a^ Bivariate binary regression showing the association between GCP-S and the mortality during hospitalization due to severe TBI in the present study.

The frequency of loss of pupil reactivity increased with decreasing GCS score: 2.07% at GCS scores 7 to 8 had a bilateral loss of pupil reactivity, 6.75% at GCS scores 5 to 6, and 21.3% at GCS scores 3 to 4. In the patients with the GCS scores 4, 5, and 6, unilateral loss of pupil reactivity occurred at similar rates: 49, 48, and 46%, respectively. Bilateral pupil reactivity was more frequent among patients with a GSC score 8 (67.8%) and less frequent in patients with a GCS score 4 (27.8%) (data not shown).

The relationship between the combined GCS-P and mortality at discharge is shown in Table 3 . The combined score extended the range over which the differentiation of outcomes was made, with the highest mortality rate of 50% in the lowest GCS score (score 3) and rising to 86% in the GCS-P in the present study. The nonmonotonic relationship between GCS and mortality, where higher mortality was observed for the GCS score of 4 rather than 3 in bivariate analysis, is no longer seen for the GCS-P score. The same holds for the relationship between the GCS-P and mortality at 6 months since severe TBI with the CRASH/IMPACT data, where the highest mortality rate increased from 51.0% to 74.4% ( Table 3 ).

**Figure 1:**
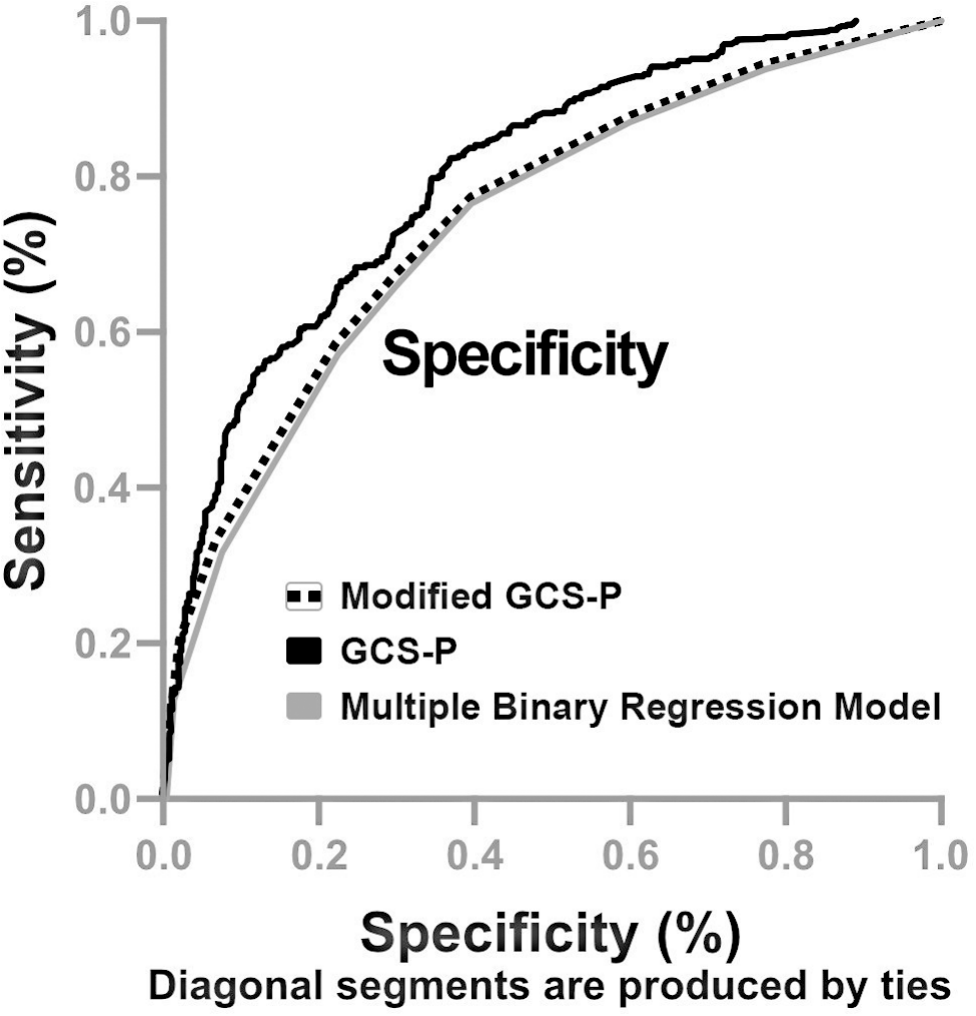
Comparison between the receiver operator characteristic (ROC) curves and their accuracy to predict hospital mortality in severe traumatic brain injury by three models: the Glasgow Coma Scale Pupil (GCS-P) score, modified GCS-P score, and the multivariate binary regression model including age, blood glucose, Marshall's cranial computed tomography classification, GCS score, pupil reactivity, and the presence of traumatic subarachnoid hemorrhage on admission. The area under the ROC was 0.73 (0.70–0.77) for the GCS-P model, 0.74 (95% confidence interval [CI]: 0.71–0.77) for its modified version, and 0.80 (95%CI: 0.77–0.83) for the model that included additional clinical and radiological variables.

The GCS-P and its modified version (Modified GCS-P) were compared in terms of simple arithmetical scores and by adding the clinical and radiological variables shown in Table 2 . The AUROC was 0.73 (0.70–0.77) for the GCS-P model, 0.74 (95%CI: 0.71–0.77) for its modified version, and 0.80 (95%CI: 0.77–0.83) for the model that included additional clinical and radiological variables ( Figure 1 ). These accuracy findings for mortality of the GCS-P score and the model combining other variables (GCS, pupil reactivity, age, cranial CT findings) were the same observed in the subgroup of patients with severe TBI from the CRASH data bank. 15

## DISCUSSION

The present work is the largest prospectively acquired database about severe TBI in Brazil and the first investigating the GCS-P score accuracy on a population level in this country. The data collected using a protocol created by the same group of researchers and the neurosurgical team involved with the patient's care aided the internal validity of the study. The results obtained align with the current literature, 10 15 24 and demonstrated that old age, CT findings, GCS, and pupil reactivity at admission are independently associated with severe TBI patient mortality during hospitalization.

According to the IMPACT and CRASH studies, severe TBI patient mortality was 33.9% within 6 months of injury. 15 18 19 This figure is likely higher for the present study patients as they reached 32.8% mortality already at discharge was 32.8%, although exact data were not available in the present study because of a limited follow-up period.

Separately, GCS score and pupil response were each related to adverse outcomes in various studies. 11 18 27 The mortality at discharge in patients with mydriatic pupils was 11 times higher than in patients with isochoric pupils in the present study – a result much higher than most TBI studies that have found about a threefold increase of this risk. 15 17 18 28 29

The difference may be due to the higher severity of the injuries among the present study patients, as almost half of them scored 3 or 4 on the GCS. However, the AUROC comparison for mortality at discharge between the GCS-P score and its modified version where mydriatic pupils scored 3, showed no statistically significant difference. The paradox is that patients with GCS score 3 had lower mortality than those with score 4 ( Table 2 ) have been reported in other studies, 29 30 31 but the reason for this is unclear. As discussed by Brennan et al., 15 this may result from allocating a score to patients whose responsiveness was depressed pharmacologically. Smoothing out of the relationship between the score and hospital mortality due to severe TBI is a further advantage of the GCS-P score. 32

Although survival prognosis based on statistical methods that combine information about multiple aspects of the condition of the TBI patient have greater accuracy, these have not found widespread acceptance in clinical practice because of their complexity. 33 The multiple binary regression model created for the present study using clinical and radiological variables ( Table 2 ) showed slightly better accuracy than the GSC-P score and the modified GSC-P score ( Figure 1 ). However, simple scoring systems for stratifying the TBI severity have been used by clinicians because of their simplicity and transparency of the score calculation. 15 30 The GSC-P score possesses these qualities and can be applied in clinical practice with an accuracy of 73% ( Figure 1 ). The proportion of deaths predicted by the GCS-P score applied to the IMPACT/CRASH data was equivalent to that of the present study ( Table 3 ), thus suggesting that this method may be suitable for predicting severe TBI mortality. Also, the GCS-P maintained an inverse relationship between the GCS-P and adverse outcomes across the complete range of all possible scores.

To conclude, the present study supports other external validation studies by showing that the GCS-P score has greater accuracy for predicting hospital mortality among severe TBI patients than GCS or pupil reactivity evaluation alone, and only slightly inferior accuracy than more complex predictive models. 15 The role of the GCS-P in predicting long-term functional outcomes, including psychiatric symptoms, cognitive performance, and quality of life, deserves further investigation.
